# Novel Approach to Repairing a Traumatic Aortic Arch Pseudoaneurysm Following a Fall

**DOI:** 10.1055/s-0039-1687902

**Published:** 2019-07-22

**Authors:** Azhar Hussain, Pouya Youssefi, Gopal Soppa, Marjan Jahangiri

**Affiliations:** 1 Department of Cardiothoracic Surgery, St. George's Hospital, London, United Kingdom

**Keywords:** aorta, pseudoaneurysm, traumatic aneurysm

## Abstract

Traumatic pseudoaneurysms of the aortic arch are often treated with surgical repair regardless of the lesion size or age. The authors report a simple, less invasive surgical repair in a patient who sustained blunt aortic injury following a fall.

## Introduction

Blunt trauma to the aortic arch can occur during road traffic accidents and falls from a significant height. These injuries are often due to a combination of direct compressive effects and indirect acceleration–deceleration forces placed on the aortic wall. The aortic isthmus is often the region where the greatest strain occurs, at the point of insertion of the ligamentum arteriosum, where up to 95% of ruptures occur. 1 2 Repair using prosthetic grafts to replace the affected segment of the aorta has been the recommended surgical approach. 3 4

## Case Presentation

**Fig. 1:**
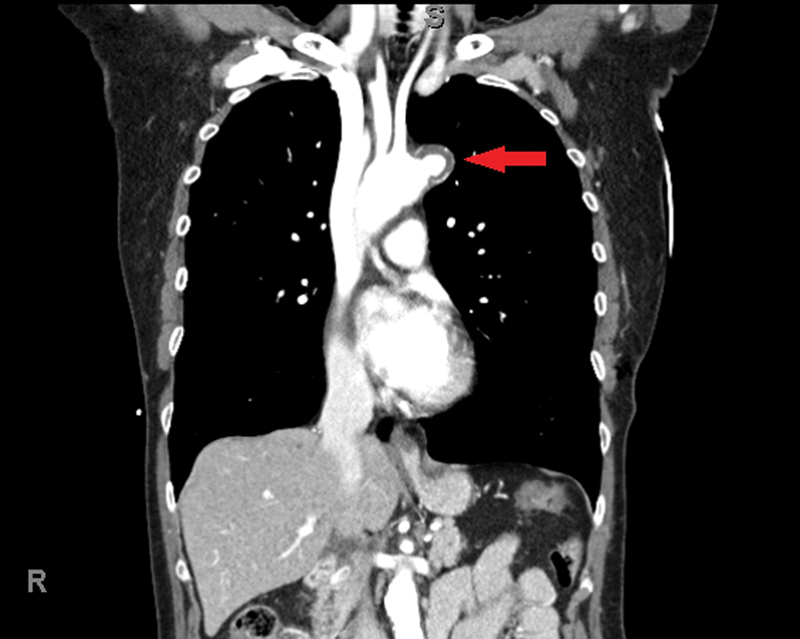
Computed tomography (CT) aorta showing the pseudoaneurysm.

A 73-year-old lady presented with a fall down of 13 stairs at her home while intoxicated. Her past medical history was significant for chronic obstructive pulmonary disease and degenerative lumbar spinal disease. She was a lifelong smoker who was independent in her daily activities. She presented to her local hospital with symptoms of neck pain and stiffness but denied any chest pain. She was hemodynamically stable and underwent a computed tomography (CT) scan. The scan revealed no obvious vertebral fractures but did reveal a focal pseudoaneurysm along the left lateral wall of the aortic arch between the origins of the left common carotid and subclavian arteries ( Fig. 1 ). This measured 19 mm in maximum anteroposterior dimension. She was subsequently referred to the vascular surgical team, who agreed to manage her conservatively with yearly CT scans.

Eleven months later, the patient represented to her local hospital with sudden onset of chest pain radiating to her back. Initial observations were unremarkable. A repeat CT of the aorta demonstrated the aneurysm with no significant size increase compared with the previous CT scan. The vascular surgical team felt that an endovascular approach would not be suitable owing to the proximity of the lesion to the head and neck vessels. Transthoracic echocardiogram and coronary angiography were normal.

**Fig. 2:**
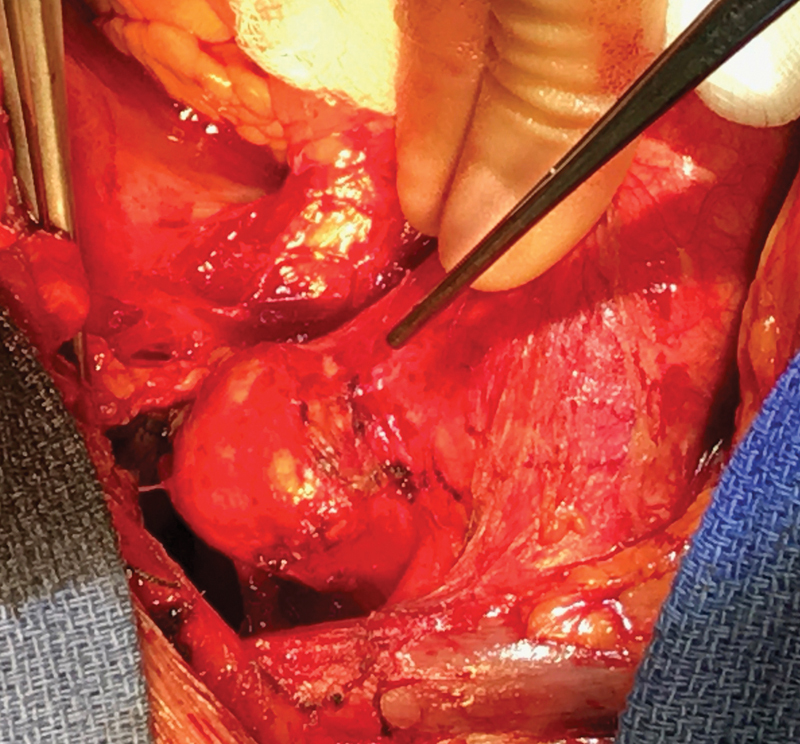
Operative picture demonstrating the pseudoaneurysm.

**Fig. 3:**
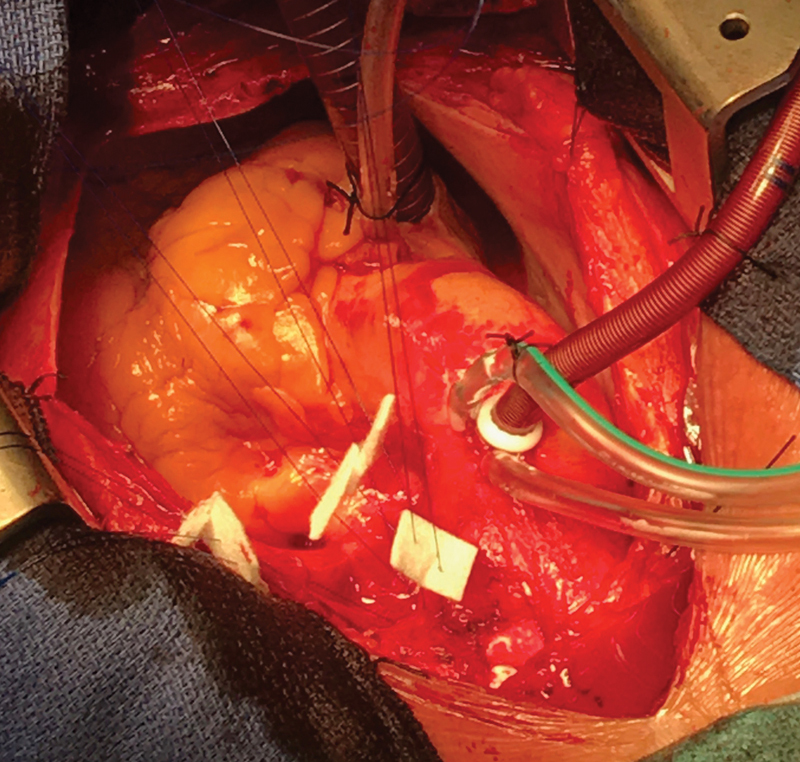
Pledgeted mattress sutures used to close the base of the aneurysm.

**Fig. 4:**
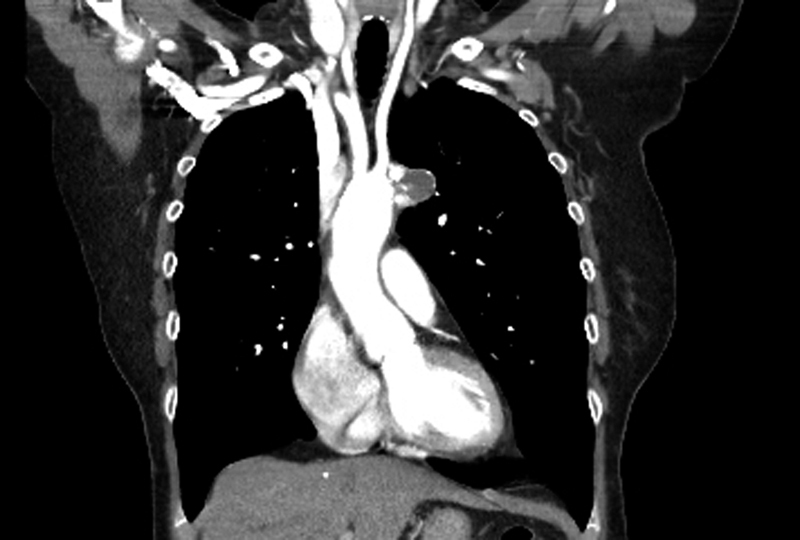
Follow-up computed tomography (CT) aorta showing no contrast filling into the aneurysm.

The patient underwent surgery via a median sternotomy. The aortic arch and the head and neck vessels were dissected. The pseudoaneurysm was identified and appeared isolated to the greater curve on the left lateral aspect of the arch ( Fig. 2 ). Following full heparinization, cardiopulmonary bypass was established by cannulation of the ascending aorta and the right atrium at a temperature of 35°C. The base of the aneurysm was approximated using several 3/0 prolene pledgeted mattress sutures from the outside, with reduced flow facilitating the closure ( Fig. 3 ). A needle was inserted into the excluded part of the aneurysm to ensure there was no persistent flow. The bypass time was 27 minutes. The patient's postoperative course was unremarkable. She was extubated on the same day and discharged home on the 5th postoperative day. She was reviewed in the outpatient follow-up clinic for 6 months and then at 1 year postoperatively. She was doing very well with no chest or back pain. A repeat CT scan at 6 months revealed an isolated saccular aneurysm which no longer filled with contrast ( Fig. 4 ). The ascending aorta and descending thoracic aorta were normal. A follow-up transthoracic echocardiogram revealed normal biventricular function and a normal sized aortic root.

## Discussion

We have shown a simple and less invasive closure of a traumatic aortic pseudoaneurysm. To the best of our knowledge, this approach is not reported elsewhere in the literature. The traditional approach to repairing a traumatic aortic pseudoaneurysm of the arch typically involves deep hypothermic circulatory arrest which is associated with a high mortality and morbidity rate (20–30%) in acute traumatic cases. 5 Our approach also significantly reduces the cardiopulmonary bypass time and its associated risks. This case demonstrates that with careful surgical planning, an uncomplicated aortic pseudoaneurysm can be treated less invasively while reducing the risks associated with the conventional open aortic approach.
